# Precuneus-Dominant Degeneration of Parietal Lobe Is at Risk of Epilepsy in Mild Alzheimer's Disease

**DOI:** 10.3389/fneur.2019.00878

**Published:** 2019-08-22

**Authors:** Andras Horvath, Mate Kiss, Anna Szucs, Anita Kamondi

**Affiliations:** ^1^Department of Neurology, National Institute of Clinical Neurosciences, Budapest, Hungary; ^2^Department of Anatomy Histology and Embryology, Semmelweis University, Budapest, Hungary; ^3^Department of Neurology, Semmelweis University, Budapest, Hungary

**Keywords:** Alzheimer's disease, epilepsy, seizure, neuroimaging, precuneus, parietal lobe

## Abstract

**Introduction:** Alzheimer's disease (AD) is the leading cause of cognitive decline. Epilepsy is a frequent comorbid condition of AD. While previous studies analyzed the risk factors of AD-related epileptic seizures, we still lack biomarkers of epilepsy in mild AD cases.

**Purpose:** The aim of our study was to analyze the correlations between neuropsychology, cortical thickness, and brain volumetric measurements in mild Alzheimer patients with concomitant epileptic seizures.

**Materials and methods:** We selected mild AD patients from our database to examine them with structural magnetic resonance imaging, 24 h electroencephalography, and detailed neuropsychology. We made the diagnosis of epilepsy based on epileptology data including neurophysiology. We retrospectively analyzed the neuropsychology pattern, clinical and epidemiologic features, cortical thickness, and volumetric values of mild AD patients with and without overt clinical seizures using covariance weighted general linear model.

**Results:** We found epileptic seizures in 26% of mild AD patients. Patients with seizures performed worse in visuo-spatial scores than patients without (*p* = 0.003). Patients with seizures had smaller parietal thickness (*p* = 0.018), being associated to reduced thickness of left (*p* = 0.007), and right precunei (*p* = 0.005). The visuo-spatial performance positively and strongly correlated with the thickness of the parietal lobe (*r* = 0.67; *p* = 0.002) and with the volume of the precuneus (*r* = 0.612; *p* = 0.005).

**Conclusion:** Epileptic seizures are common even in mild AD. We found that a prominent deficit in visuo-spatial skills is a red flag for epileptic seizures in the initial phase of AD, indicating the early involvement of parietal lobe in the neurodegenerative process. Because our findings suggest that the degeneration of precuneus is a sensitive marker of seizures associated to mild AD, clinicians need to pay special attention to the pattern of atrophy shown by structural MRI. Our results confirm previous data suggesting that epileptic seizures might be associated to a faster progressing type of AD with the early degeneration of posterior cortical areas.

## Introduction

Alzheimer's disease (AD) is the major cause of cognitive decline, creating tremendous economic burden on the aging societies. The main symptoms of AD are loss of episodic memory and orientation; the impairment of social, behavioral, and communication skills. Since we cannot significantly affect the progression of cognitive deterioration, increasing number of studies have examined the potentially modifiable risk factors and concomitant conditions of AD.

Epilepsy is a frequent comorbidity of dementia. In animal models of AD, epileptic seizures and epileptiform activity are frequently presented (1, 2). Neuropathological studies have identified many similarities between AD and epilepsy, e.g., amyloid plaques, tau neuro-fibrils, hippocampal sclerosis, and hyperexcitability of the hippocampus (3–6).

Growing body of evidence suggests that AD patients have an increased risk to develop epileptic seizures (7). Although the results of early prevalence studies on seizure frequency are variable, recent studies using more sensitive diagnostic tools have revealed a high prevalence of seizures. Rauramaa et al. identified seizures in 17% of 64 neuropathologically confirmed AD cases (8) and Zarea et al. reported seizures in 19% of presenilin-1, in 28% of presenilin-2, and in 31% of amyloid precursor protein genetic mutation carriers (9). Our group identified seizures in 24% of AD patients using long-term EEG monitoring (10). Based on these results, recent reviews agree that epileptic seizures show high incidence in AD (10–13).

Risk analysis studies found that early onset of cognitive decline is a consistent risk factor for epileptic seizures in AD (8, 14, 15). Advanced disease stage as a risk factor has been also demonstrated (10, 14, 16); however, seizures occur in the earlier phases of AD as well (17). Detection of patients with seizures could be challenging in initial AD, since the mentioned risk factors have been extracted from large samples containing patients in various disease stages (18). Unfortunately, we also lack studies on cerebrospinal fluid biomarkers for the prediction of seizures in AD (18). Diagnosis of seizures based on witness accounts and interviews may be insufficient: non-motor temporal seizures may be hard to detect especially in permanently confused patients (10, 19). Routine EEG is also unreliable in the detection of epileptic activity in AD (20). Long-term EEG and magnetoencephalography represent trustable diagnostic tools; however, they are rarely available expensive techniques and their interpretation requires specific experience and expertise (21, 22).

To our best knowledge, no studies have analyzed the relation of neuroimaging features and epileptic activity in AD, although seizures and epileptiform activity might be associated to accelerated progression of dementia (22) and the treatment of seizures might have beneficial impact on the life quality of AD patients (23).

Because of converging paths between epilepsy and AD (24) and the higher mortality of AD patients with seizures (25), we aimed to study the utility of structural brain magnetic resonance imaging in the signaling of seizures in mild phase of AD.

## Materials and Methods

### Subjects

We analyzed the data of 73 AD patients included in the Alzheimer-Epilepsy Project at the Department of Neurology of National Institute of Clinical Neurosciences in Budapest between 2014 and 2017. The patients met the revised diagnostic criteria of the National Institute on Aging-Alzheimer's Association (NIA-AA) for probable AD (26). Patients presenting epileptic seizures preceding the onset of cognitive decline were excluded from the study. Known seizure-provoking factors were also used as exclusion criteria: history of central nervous system infection, head trauma with loss of consciousness, stroke, major depression, schizophrenia, electroconvulsive therapy, any chronic medical condition, and alcohol or drug dependency. Since we have no access to PET biomarkers and limited access to CSF biomarker analysis in our country, we selected the bitemporal, bifrontal, and hippocampal atrophy on the structural MRI as supporting biomarkers; however, it represents a limitation for our study (26). MRI lesions other than the typical features of AD were not permitted (27). Patients underwent detailed physical, neurological, and epileptology examination, as well as routine blood checks. We retrospectively collected data of past medical history from both patients and relatives and scrutinized medical records with special attention to epileptology considerations.

### Neuropsychology

Neuropsychology testing was carried out by trained neurologists or neuropsychologists. We used the Addenbrooke Cognitive Examination (ACE) as the primary test battery because of its high sensitivity and specificity in the diagnosis of dementia. ACE is an accurate tool in differentiating frontotemporal dementia and AD, and it serves properly in the assessment of dementia severity with the extraction of Mini-Mental State Examination Score (MMSE) from the test values (28). MMSE is widely accepted in determining dementia severity stages. Scores between 26 and 30 indicate normal cognition; 21–25, mild dementia; 11–20, moderate dementia; and 0–10, severe dementia (29). Since anxiety and depressive symptoms might compromise cognitive functions, we also recorded Spielberger State and Trait Anxiety Inventory (STAI) and Beck Depression Inventory II (BDI-II). Based on large samples, <45 value on STAI represents low level of anxiety in both the trait and state category (30). BDI scores <13 indicate minimal depression, the 14–19 range represents a mild one, the range 20–28 is a moderate one, and a score >29 marks severe depression (31). To increase our diagnostic accuracy, patients with a STAI > 45 and a BDI II > 13 were excluded from this analysis. The National Ethic Committee of Hungary authorized our research (024505/2015/OTIG). We obtained an informed and written consent from each patient.

### Neurophysiology

For neurophysiology assessment, we used 24 h Holter-EEG with 34 channels [Micromed Morpheus Polysomnograph, 10–20 electrode placements; frontal (Fp1, Fp2, F3, F4), frontocentral (Fz), central (C3, Cz, C4), centroparietal (Pz), frontotemporal (F7, F8), temporal (T3, T4, T5, T6), parietal (P3, P4), and occipital (O1, O2) electrodes]. EEGs were recorded at the time of diagnosis of AD. Epileptiform discharges were defined as paroxysmal EEG graphoelements (spikes or sharp waves) with 20–200 ms duration, with the disruption of background EEG activity, followed by slow waves (32). Two independent raters analyzed the EEGs; a graphoelement was considered as epileptiform if both raters defined it so. Epilepsy was diagnosed based on the current guidelines: two independent seizures or one seizure with a high chance for reoccurrence (33). Patients with one seizure and epileptiform potentials interictally on EEG were diagnosed with epilepsy.

### Neuroimaging

All subjects were examined using brain MRI producing high-resolution structural images being used for further processing and analysis. All data were acquired on a Siemens Magnetom Verio 3T MRI scanner (Siemens Healthcare, Erlangen, Germany) at the National Institute of Clinical Neurosciences, Budapest, Hungary. The standard 12-channel head receiver coil was applied. The protocol consisted of T1-weighted 3D MPRAGE (magnetization prepared rapid gradient echo) anatomical imaging (TR = 2,300 ms; TE = 3.4 ms; TI = 1,100 ms; flip angle = 12°; voxel size: 1.0 × 1.0 × 1.0 mm). Cortical reconstruction and volumetric segmentation were performed by Freesurfer 6.0 image analysis suite being documented and freely available for download online (http://surfer.nmr.mgh.harvard.edu/). The technical details of these procedures are demonstrated in previous reports; we did not change this pipeline and used the “recon-all” processing stream with the default parameters to form a cortical surface model. Particularly, image processing involves motion correction, removal of non-brain tissue using a hybrid watershed/surface deformation process, automated Talairach transformation, segmentation of the subcortical white matter and deep gray matter volumetric structures (including hippocampus, amygdala, caudate, putamen, and ventricles), intensity normalization, tessellation of the gray matter white matter boundary, automated topology correction, and surface deformation following intensity gradients to optimally place the gray/white and gray/cerebrospinal fluid borders at the location where the greatest shift in intensity defines the transition to the other tissue class (34). When the cortical models were finished, Freesurfer performed numerous deformable processes for following data processing and analysis. Steps included surface inflation, registration to a spherical atlas using individual cortical folding patterns to match cortical geometry across subjects, parcellation of the cerebral cortex into units based on gyral and sulcal structure, and creation of a variety of surface-based data including maps of curvature and sulcal depth. Finally, cortical models and the results of segmentation were quality checked and manually corrected on each 20 subject.

### Statistical Analysis

Differences in epidemiologic features and clinical characteristics across study groups (AD patients with and without overt epileptic seizures) were compared with two-tailed *t*-tests and chi-square tests. General linear model (GLM) was applied for intergroup comparisons on brain volumes and cortical thickness with age, sex, disease duration, and disease severity (emphasized in ACE score) as covariates. Volumetric measurements were performed in brain areas traditionally connected to AD: hippocampus, entorhinal cortex, fusiform gyrus, precuneus, parahippocampal gyrus, posterior cingulate gyrus, and amygdala (34–37). Cortical thickness was analyzed as an average of specific cortical areas of different lobes (Table 1). Significant difference was defined as *p* < 0.05. Where significance (*p* < 0.05) was found, we analyzed further the lobar pattern regarding the various lobar areas. The connection between the volume and thickness of brain areas and the results of neuropsychology was analyzed by Pearson correlation. Effect sizes were emphasized in Hedges' *g* in parametric comparisons and in Fisher's *z* in chi-square analysis. Effect size <0.3 was considered as small, 0.3–0.6 as medium, and >0.6 as large according to Cohen.

**Table 1:** The calculated cortical thickness values.

**Parameter**	**Cortical contributors**
Mean frontal thickness	Superior frontal gyrus, middle frontal gyrus, opercular part of inferior frontal gyrus, triangular part of inferior frontal gyrus, orbital part of inferior frontal gyrus, precentral gyrus, lateral orbitofrontal area, medial orbitofrontal area, paracentral lobule, frontal pole
Mean parietal thickness	Precuneus, superior parietal lobule, inferior parietal lobule, supramarginal gyrus, postcentral gyrus
Mean temporal thickness	Superior temporal gyrus, middle temporal gyrus, inferior temporal gyrus, parahippocampal gyrus, fusiform gyrus, entorhinal area, transverse temporal area, temporal pole, banks of superior temporal sulcus
Mean occipital thickness	Lateral occipital gyrus, lingual gyrus, cuneus, pericalcarine area
Mean cingulate thickness	Posterior cingulate cortex, rostral anterior cingulate cortex, caudal anterior cingulate cortex, isthmus of cingulate cortex

## Results

### Epidemiology, Clinical, and Neuropsychology Data

**Figure 1:**
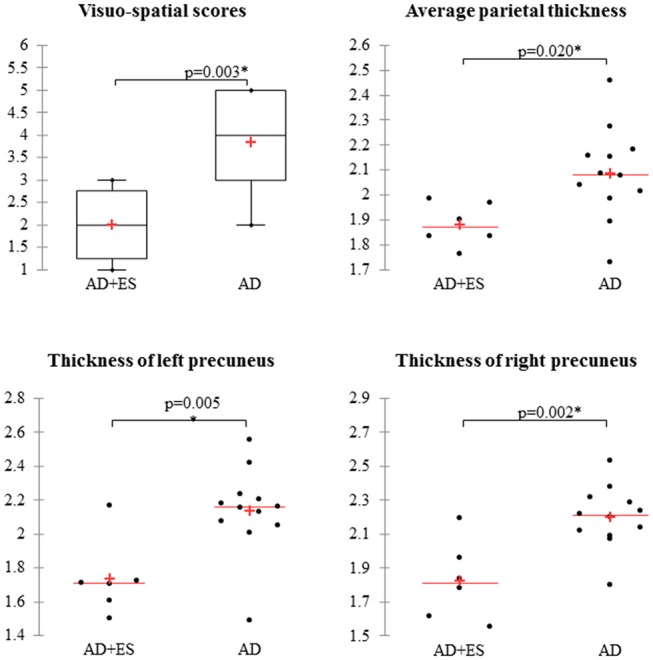
Mild Alzheimer patients with epileptic seizures show significantly impaired visuo-spatial skills and decreased average parietal thickness, mainly reduced thickness of precunei compared to AD patients without epilepsy. AD, Alzheimer's disease; ES, epileptic seizures. *Significant difference, *p* < 0.05.

After the exclusion of 46 patients with moderate and severe AD, 27 mild AD patients were included for further analysis. We found epileptiform discharges on the EEG in 7 out of 27 patients without overt clinical seizures. These patients have been excluded as well from statistics, since epileptiform discharges might be accidental EEG findings in AD without direct connection to seizure activity (subclinical epileptiform activity) (10, 38). Finally, we involved 20 mild AD patients for further analysis. Seven patients had epileptic seizures confirmed by interictal epileptiform discharges as well. In 13 patients, we did not find seizures or epileptic activity on the EEG recordings. Thus, the prevalence of epileptic seizures was 25.9% in our mild AD cohort (7/27), while the EEG showed epileptiform discharges in 51.85% (14/27). Epileptiform discharges were visible on temporal electrodes with maximum electronegativity, namely, on bilateral temporal channels (*n* = 3), on left temporal channels (*n* = 7), and on right temporal channels (*n* = 4). One patient suffered generalized tonic–clonic seizures, and six additional patients reported focal seizures with temporal semiology, namely, recurrent short episodes with impaired awareness (e.g., memory loss and episodic confusion). Based on our previous experience and scientific reports (7, 12, 17), all the patients were treated with 2 × 500 mg/day levetiracetam (LEV). LEV proved to be well tolerated, and we reached complete seizure control by all patients. Epidemiology and clinical and neuropsychology data of the two patient groups (AD without epileptiform discharges or epileptic seizures = AD; AD with epileptic seizures and interictal discharges = AD+ES) are shown in Table 2. We have not revealed significant differences across the study groups in almost any category; however, patients with epileptic seizures had a significantly more severe impairment in visuo-spatial skills (*p* = 0.003) with a high effect size (1.49) (Table 2, Figure 1). In the AD+ES group, females were significantly overrepresented (*p* = 0.023) with low effect size (0.22).

**Table 2:** Epidemiologic and clinical data of our patients.

**Parameter**	**AD+ES**	**AD**	***p*-value of *t* and χ^**2**^ tests**	**Effect size in Hedges' *g* and in Fisher's *z***
Number of patients	7	13	-	-
**Gender (number of females)**	**5/7**	**5/13**	*0.023	**0.22**
Memantine therapy (% of patients)	0%	0%	1	-
Cholinesterase inhibitor therapy (% of patients)	100%	100%	1	-
Age (years)	72.1 ± 3.8	71 ± 8.4	0.366	0.15
Age at the onset of dementia (years)	66.7 ± 3.1	68.4 ± 7.9	0.488	0.25
Duration of dementia (years)	3.1 ± 1.6	2.6 ± 1.8	0.597	0.28
Number of education years	15 ± 1.7	15.4 ± 1.7	0.412	0.23
Age at first seizure (years)	70.3 ± 3.9	-	-	-
Seizure frequency (/month)	1.7 ± 0.7	-	-	-
Antiepileptic medication (daily dose)	2 × 500mg LEV	-	-	-
MMSE score (range: 0–30)	24.4 ± 0.5	24.3 ± 0.4	0.1	0.26
ACE score (range: 0–100)	72.7 ± 4.9	76.3 ± 6	0.512	0.63
VLOM ratio (FTD < 2.2, normal: 2.2–3.2; 3.2 < AD)	3.4 ± 0.2	3.3 ± 0.1	0.182	0.7
Orientation (range: 0–10)	8.4 ± 0.9	8.5 ± 0.9	0.086	0.11
Attention (range: 0–8)	6.6 ± 0.7	6.6 ± 0.8	0.11	0
Memory (range: 0–35)	22.3 ± 4.8	24.7 ± 4.4	0.58	0.52
Verbal fluency (range: 0–14)	10.6 ± 1.6	10.3 ± 1.5	0.771	0.19
Language (range: 0–28)	22.9 ± 2.7	21.8 ± 2.9	0.468	0.38
**Visuo-spatial skills (range: 0–5)**	**2.3** **±** **1**	**3.9** ± **1.1**	*0.003	**1.49**
STAI-S (range: 20–80)	31.6 ± 6.6	32.6 ± 3.9	0.897	0.2
STAI-T (range: 20–80)	32.3 ± 4.1	31.4 ± 4.4	0.475	0.2
BDI (range: 0–30)	7.7 ± 1.5	7.1 ± 3.2	0.476	0.21

AD, Alzheimer's disease; ES, epileptic seizure; ^*^Significant difference, p < 0.05. LEV, levetiracetam; MMSE, Mini-Mental Score Examination ranging between 0 and 30; ACE, Addenbrooke Cognitive Examination score ranging between 0 and 100; VLOM ratio, represents the ratio of verbal fluency and language performance vs. the retrograde memory and orientation points; FTD, frontotemporal dementia; STAI-S, State–Trait Anxiety Inventory State score ranging between 20 and 80; STAI-T, State–Trait Anxiety Inventory Trait score ranging between 20 and 80; BDI, Beck Depression Inventory ranging between 0 and 30.

### Differences in Cortical Volumes and Thickness Between Mild AD Patients With and Without Epileptic Seizures

**Figure 2:**
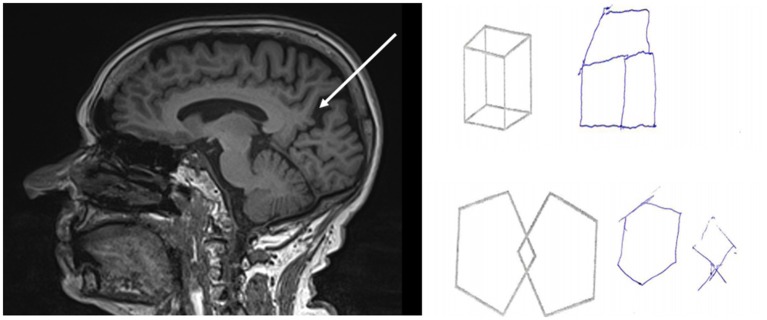
Results of brain MRI and neuropsychology testing of a mild Alzheimer patient with epileptic seizures. Figure demonstrates the impaired visuo-spatial skills examined with Addenbrooke Cognitive Examination and visible precuneal degeneration on MRI with deep sulci surrounding the precuneus (white arrow).

We found significant difference in parietal thickness across the study groups (*F* = 4.018; *p* = 0.018) with high effect size (1.41) (Table 3). Patients with epileptic seizures showed significantly smaller parietal thickness (Figure 1). Age (*F* = 1.065; *p* = 0.32), sex (*F* = 0.001; *p* = 0.97), disease duration (*F* = 0.099; *p* = 0.758), or disease severity (*F* = 0.139; *p* = 0.716) did not have significant effect on parietal thickness. Since parietal lobe atrophy differed significantly across the groups, we further analyzed the structures of parietal lobe. We found that precuneal thickness variety was responsible for the parietal difference, since both left (*F* = 5.116; *p* = 0.007) and right precunei (*F* = 5.629; *p* = 0.005) differed across the study groups with high effect sizes (1.81 for left, 2.09 for right), while other parts of the parietal lobe were not significantly different (Table 4). Covariates did not have a significant effect on left (age: *F* = 2.674, *p* = 0.0124; sex: *F* = 0.091, *p* = 0.767; disease duration: *F* = 0.729, *p* = 0.407; disease severity: *F* = 1.434, *p* = 0.251) or on right precuneal thickness (age: *F* = 0.553, *p* = 0.47; sex: *F* = 0.079, *p* = 0.783; disease duration: *F* = 0.116, *p* = 0.739; disease severity: *F* = 3.171, *p* = 0.097). Precuneal thickness was significantly smaller in the AD+ES group (Figure 2).

**Table 3:** Results of age-, sex-weighted ANOVA in the comparisons of brain volumes and cortical thickness across Alzheimer's disease group (AD) and the Alzheimer group with epileptic seizures (AD+ES).

**Structure**	**AD+ES (mean ± *SD*)**	**AD (mean ± *SD*)**	***F*-value of ANOVA**	***p*-value of ANOVA**	**Effect size in Hedges' *g***
**Volumes (mm**^**3**^**)**					
Hippocampi	3496 ± 403	3683 ± 553	0.687	0.64	0.36
Entorhinal cortex	1921 ± 444	2039 ± 292	0.63	0.68	0.33
Fusiform gyri	8039 ± 1338	8417 ± 1668	0.208	0.953	0.24
Precuneus	7411 ± 1419	8181 ± 1410	0.834	0.547	0.53
Parahippocampal gyri	1750 ± 351	1920 ± 339	1.125	0.392	0.49
Posterior cingulate gyri	2345 ± 495	2501 ± 952	0.626	0.683	0.18
Amygdaloid bodies	1379 ± 267	1608 ± 352	0.554	0.773	0.7
**Cortical thickness (mm)**					
Average frontal	2.2 ± 0.29	2.27 ± 0.21	0.156	0.975	0.29
**Average parietal**	**1.85** ± **0.12**	**2.08** ± **0.18**	**4.018**	^*^0.018	**1.41**
Average temporal	2.51 ± 0.38	2.54 ± 0.28	0.202	0.956	0.09
Average occipital	1.73 ± 0.18	1.75 ± 0.18	0.012	0.312	0.11
Average cingular	2.26 ± 0.32	2.37 ± 0.19	0.039	0.597	0.45

* *Significant difference, p < 0.05*.

**Table 4:** Results of age-, sex-weighted ANOVA comparing cortical thickness of parietal lobe structures across Alzheimer's disease group (AD) and the Alzheimer group with epileptic seizures (AD+ES).

**Cortical thickness of parietal structures (mm)**	**AD+ES (mean ± *SD*)**	**AD (mean ± *SD*)**	***F* value**	***p*-value**	**Effect size in Hedges' *g***
**Left precuneus**	**1.71** **±** **0.21**	**2.14** **±** **0.25**	**5.116**	^*^0.007	**1.81**
**Right precuneus**	**1.79** **±** **0.24**	**2.20** **±** **0.17**	**5.629**	^*^0.005	**2.09**
Left superior parietal lobule	1.79 ± 0.14	2.00 ± 0.27	1.106	0.401	0.89
Right superior parietal lobule	1.79 ± 0.11	1.99 ± 0.23	1.150	0.381	1
Left inferior parietal lobule	1.96 ± 0.32	2.17 ± 0.31	0.87	0.525	0.67
Right inferior parietal lobule	2.00 ± 0.33	2.21 ± 0.25	0.785	0.577	0.75
Left supramarginal gyrus	1.97 ± 0.35	2.17 ± 0.25	0.717	0.621	0.69
Right supramarginal gyrus	1.95 ± 0.22	2.21 ± 0.28	2.061	0.132	0.99
Left postcentral gyrus	1.76 ± 0.15	1.85 ± 0.15	1.415	0.278	0.6
Right postcentral gyrus	1.76 ± 0.16	1.83 ± 0.19	0.477	0.787	0.39

* *Significant difference, p < 0.05*.

### Correlation Analysis Across Structural Features and Neuropsychology Results

Significant positive correlation was found between the ACE scores and the volumes of temporal lobe structures with a predominance of entorhinal volume (*r* = 0.68; *p* = 0.001) and the volume of fusiform gyrus (*r* = 0.64; *p* = 0.03). ACE score was also highly correlated to average frontal and parietal thickness (frontal: *r* = 0.51; *p* = 0.024; temporal: *r* = 0.646; *p* = 0.003), while the correlation with other lobes was not significant. Visuo-spatial score showed highest positive correlation to precuneal volumes (*r* = 0.612; *p* = 0.005), while positive correlation was demonstrated to temporal structures as well. It was also correlated to average parietal thickness with the highest values (*r* = 0.67; *p* = 0.002) and to temporal thickness with lower values (*r* = 0.41; *p* = 0.034). Correlation to other cortical lobes was not significant (Table 5).

**Table 5:** Results of Pearson correlation across the brain volumes, cortical thickness, and Addenbrooke Cognitive Examination (ACE) score and visuo-spatial scores.

**Structure**	**Correlation with ACE score (*r*; *p*-values)**	**Correlation with visuo-spatial results (*r*; *p*-values)**
**Volumes (mm**^**3**^**)**		
Hippocampi	^*^(0.546; 0.016)	^*^(0.41; 0.08)
Entorhinal cortex	^*^(0.68; 0.001)	^*^(0.52; 0.022)
Fusiform gyri	^*^(0.64; 0.03)	^*^(0.53; 0.018)
Precuneus	(0.39; 0.19)	^*^(0.612; 0.005)
Parahippocampal gyri	^*^(0.45; 0.05)	^*^(0.52; 0.02)
Posterior cingulate gyri	(0.18; 0.44)	(0.18; 0.45)
Amygdaloid bodies	^*^(0.52; 0.022)	(0.13; 0.59)
**Thickness (mm)**		
Average frontal	^*^(0.51; 0.024)	(0.19; 0.43)
Average parietal	(0.038; 0.878)	^*^(0.67; 0.002)
Average temporal	^*^(0.646; 0.003)	^*^(0.41; 0.034)
Average occipital	(0.24; 0.304)	(0.175;0.47)
Average cingular	(0.46; 0.074)	(0.07; 0.77)

* *Significant difference, p < 0.05*.

## Discussion

A growing body of evidence recognizes that Alzheimer patients have a high chance to develop epileptic seizures (7). While treatment of seizures is essential to provide a better quality of life, precise recognition of epilepsy is challenging due to methodological difficulties. Thus, identification of risk factors and signals of epilepsy in mild dementia is an important direction for current Alzheimer studies.

Early onset of cognitive decline is a consistent risk factor of Alzheimer-related epilepsy. In the study of Amatniek and his colleagues, early onset was associated to 0.89/year increase in relative risk (14). The same study demonstrated that more severe dementia stage was a risk as well (relative risk = 4.15). The significance of early onset is recognized in other studies as well (15, 39), while others highlighted that patients had a higher risk to develop seizures in severe dementia state (16). In our previous report (10), we described the advanced state of cognitive decline as a major risk factor of epilepsy (0.874 odds ratio for 1-point increase in ACE score). Several studies reported seizures even in the early phases of AD or in mild cognitive impairment (17, 40). In the current study, we found seizures in 26% of mild AD patients. The relatively low sample size of mild AD patients (*n* = 27) does not allow consistent conclusions on the prevalence of seizures in mild AD; however, our study confirms those reports suggesting that seizures have a high prevalence in initial cognitive impairment. In our cohort, there was no significant difference in the mean age of dementia onset in patients with seizures vs. those without but there was a clear tendency of younger onset in patients with seizures (the mean age of patients with seizures was 66.7, and the mean age of patients without seizures was 68.4). The lack of statistical significance might be related to the small sample size. We could not calculate the effect of dementia severity because a mild dementia was an inclusion criterion; however, we found a significant difference in visuo-spatial skills: patients with seizures had a lower performance, suggesting more severe impairment of parietal lobe functions. Previous reports have not analyzed the neuropsychological differences across epileptic and non-epileptic mild Alzheimer patients (18).

There are just a few studies focusing on the neuroimaging features of AD patients with epilepsy, and they do not differentiate between the stages of dementia. Previous reports highlighted more severe atrophy in AD patients with seizures without specific pattern distinction; however, the atrophy mostly correlated with the level of cognitive decline and was determined by visual analysis (14, 17, 41). In our sample, patients with seizures had no significantly higher atrophy of most lobar structures disregarding the parietal lobe. There is a clear general tendency: epileptic patients had lower volumes and thickness in all measured brain structures. The MMSE and ACE scores characterizing dementia severity correlated better with the level of atrophy both according to a previous report (34) and our findings: ACE score correlates with the atrophy of temporal and frontal lobe structures. Several studies have shown that vascular lesions increase the incidence of seizures of AD patients (14, 20, 41), but we have excluded patients with vascular lesions evidenced by MRI. Functional neuroimaging studies revealed that posterior hypometabolism shown by positron emission tomography (PET) including the temporoparietal junction, posterior cingulate gyrus, and parietal lobes might be associated to epileptic seizures in AD (42, 43). These findings correlate well with those reports suggesting that extratemporal hypometabolism or atrophy signals epilepsy in AD (44, 45). In a recent review, Cretin et al. summarize their previous experience and suggest that extratemporal representation of AD is a red flag for epileptic seizures (18).

To our best knowledge, our study is the first systematic analysis of the pattern of atrophy measured by structural MRI in mild AD patients with co-occurring epileptic seizures. Our results support the idea that epilepsy might be associated with extratemporal degeneration in AD. We found significantly reduced parietal thickness in epileptic AD patients. Interestingly, significant differences in volumes were not presented. Noticeably, previous studies reported poor correlation between volumetric data and cortical thickness (46), suggesting that cortical thickness represents more the number of surface cortical neurons, while in volumes, the deeper subcortical neurons and their surface projections are emphasized as well. It is supported by AD studies showing that reduction of cortical thickness is a more sensitive marker for the progression of dementia than volumetric changes (47). Notably, the degeneration of the precuneus was the most significant contributor to reduced cortical thickness. Interestingly, previous studies showed that the thickness of precuneus is barely affected by genetic factors, and its reduction sensitively signals pathologic processes (48). The specific pattern of atrophy is reinforced in our study by the neuropsychologic findings as well, since there is a high correlation across visuo-spatial skills and the degeneration of the precuneus. Furthermore, AD patients with seizures showed significantly lower visuo-spatial scores.

The involvement of the precuneus in epileptic seizures has not been completely clarified yet. Direct connection between the generation of seizures and the selective atrophy of the precuneus is unlikely, since these patients show temporal interictal semiology and temporal ictal pattern. Higher presence of seizures might be explained by the general importance of parietal degeneration in AD. It is widely accepted that posterior variants of AD represent a more aggressive form of AD (49, 50). These patients show initial deficit of parietal functions, earlier onset of cognitive decline, and faster progression (50). The exact reason is not clearly understood, but many studies postulate that these patients might suffer from a mixed variant of AD combining typical AD and diffuse Lewy-body pathology (51). This is in line with the reports on AD-related epilepsy demonstrating that patients with seizures show earlier onset and accelerated progression (22). Interestingly, based on studies focusing of medial–temporal lobe epilepsy, it is also possible that the more severe atrophy of the parietal lobe is a negative consequence of seizures. Neuroimaging studies revealed reduced blood flow in parietal structures during complex partial seizures (52) and a deactivation of posterior cingulate cortex following interictal discharges (53). These findings might help to explain the altered consciousness during temporal seizures; namely, many studies hypothesize that epileptic activity might deactivate the default mode network, leading to impaired cognitive functions and consciousness (54, 55). Our patients also showed focal seizures with impaired consciousness. Furthermore, it is widely accepted that decreased blood flow is associated to elevated production of pathologic AD proteins and to increased neurodegeneration (56). The connection is probably bidirectional: (1) parietal degeneration indicates a more severe pathology of AD being associated to higher incidence of seizures, and (2) epileptic activity accelerates the neurodegeneration in AD.

## Conclusion

The main limitation of our study is the small sample size; it seems that patients with mild cognitive decline seek medical help rarely or they are accidentally underrepresented in our sample. Another important limitation is the lack of CSF and PET biomarkers decreasing the homogeneity of our sample. Further studies involving higher numbers of AD patients are clearly needed to analyze the associations of the pattern of atrophy and the presence of seizures. Our results are in line with the suggestions of previous case studies: parietal lobe atrophy signals higher risk of seizures in mild AD. Proper assessment of structural neuroimaging findings and paying attention to the decreased visuo-spatial skills with detailed neuropsychology might help in the precise recognition of AD-associated epileptic seizures. Further analysis of the connection of the precuneus and AD-related seizures might lead to a better understanding of the neuropathologic process of neurodegeneration.

## Data Availability

The datasets for this manuscript are not publicly available because of the health data protection of our patients. Requests to access neuroimaging datasets should be directed to AH (email: andras.horvath.semmelweis@gmail.com).

## Author Contributions

AH conducted the neuropsychology assessments, analyzed the EEG recordings, and performed the statistical analysis. He was a major contributor in the writing of the manuscript. MK was involved in the neuroimaging data acquisition and analyzed the structural MRI findings. AS was a major figure in the recruitment of patients and in the analysis of neurophysiology recordings and contributed in the general editing of the manuscript. AK was involved in all phases as a supervisor giving important feedback for the neurophysiology assessment, for the patient recruitment, and for data acquisition and analysis. She also helped in the editing and writing of the manuscript.

### Conflict of Interest Statement

The authors declare that the research was conducted in the absence of any commercial or financial relationships that could be construed as a potential conflict of interest.
